# Association and incremental predictive performance of preoperative nutritional and inflammatory indices for postoperative ICU admission after hepatobiliary and pancreatic tumor surgery

**DOI:** 10.3389/fnut.2026.1978034

**Published:** 2026-10-22

**Authors:** Longyun Ou, Haozhu Wang

**Affiliations:** 1 Dongguan Qingxi Hospital, Dongguan, China; 2 The Seventh Clinical College of Guangzhou University of Chinese Medicine, Shenzhen, China

**Keywords:** geriatric nutritional risk index, hepatobiliary and pancreatic cancer, incremental predictive performance, nutritional and inflammatory indices, postoperative intensive care unit admission

## Abstract

**Background:**

Preoperative nutritional and inflammatory status may influence perioperative outcomes in patients undergoing hepatobiliary and pancreatic tumor surgery. This study aimed to compare the associations and incremental predictive value of six nutrition- and inflammation-related indices for postoperative intensive care unit (ICU) admission.

**Methods:**

This retrospective cohort study included 1,195 patients undergoing hepatobiliary or pancreatic tumor surgery from the INSPIRE database. We evaluated the geriatric nutritional risk index (GNRI) and five other nutritional and inflammatory indices using logistic regression and restricted cubic splines. Regression estimates were pooled across 20 imputed datasets using Rubin’s rules. Predictive metrics were averaged across imputations, with bootstrap assessment of uncertainty and optimism.

**Results:**

Among 1,195 patients, 141 (11.8%) experienced postoperative ICU admission. Higher transformed GNRI was associated with lower adjusted odds of ICU admission (OR 0.601, 95% CI 0.497–0.728). Adding GNRI increased the area under the receiver operating characteristic curve (AUC) from 0.766 to 0.800 (95% CI, 0.764–0.835). The incremental AUC was 0.033 before rounding, and the optimism-corrected AUC was 0.780 (95% CI, 0.744–0.816). Albumin alone yielded a similar apparent AUC of 0.799. Other indices added little discrimination beyond GNRI.

**Conclusion:**

GNRI showed the highest observed incremental discrimination among the six indices, although albumin alone performed similarly. These findings support further evaluation of preoperative risk assessment in hepatobiliary and pancreatic tumor surgery.

## 1 Introduction

Hepatobiliary and pancreatic cancers comprise a group of highly malignant tumors arising from the liver, biliary tract, and pancreas, encompassing diverse tissue origins, biological behaviors, and treatment pathways. Hepatocellular carcinoma, biliary tract malignancies, and pancreatic cancer contribute substantially to the global burden of disease and mortality (1). For resectable hepatobiliary and pancreatic tumors, surgical resection remains an important therapeutic approach for achieving long-term disease control. With advances in minimally invasive surgery (2–4), anesthetic monitoring (5–7), and perioperative management (8–10), the safety of hepatobiliary and pancreatic procedures has improved. However, these procedures involve complex anatomical dissection and substantial surgical stress, and patients remain at risk of postoperative hemodynamic instability (11), organ dysfunction (12), and other serious complications (13). Postoperative admission to the intensive care unit (ICU) represents an important perioperative outcome and use of critical care resources (14, 15). It may reflect planned monitoring or an unanticipated need for organ support. Therefore, identifying patients at high risk of postoperative ICU admission before surgery is clinically meaningful for developing perioperative management strategies, optimizing monitoring intensity, and allocating ICU resources appropriately.

Perioperative risk among patients with hepatobiliary and pancreatic tumors is closely associated with preoperative nutritional reserves and systemic inflammatory status. Cancer-related wasting (16), reduced food intake (17), biliary obstruction (18), impaired hepatic synthetic function (19), and pancreatic exocrine dysfunction (20) may contribute to weight loss, hypoalbuminemia, and depletion of nutritional reserves through multiple mechanisms. Systemic inflammation associated with cancer can be reflected by hematological alterations involving leukocytes, lymphocytes (21), and platelets (22), which indicate the patient’s immune-inflammatory status. Malnutrition and persistent inflammation jointly impair patients’ tolerance to surgical stress and may affect infection control and postoperative organ function recovery (23). Nutritional and inflammatory composite indices derived from routine preoperative anthropometric and laboratory parameters provide a practical approach for quantifying patients’ preoperative physiological reserves and overall systemic status.

Available nutritional and inflammatory indices combine albumin, body-size measurements, hemoglobin, and peripheral blood cell counts to characterize overlapping aspects of physiological reserve (24–29). Previous studies have mainly focused on associations between individual indices and outcomes such as mortality and hospital stay. However, an association does not establish additional predictive value beyond conventional clinical factors. Direct comparisons are needed to assess the relative discriminatory performance of these indices and whether they provide additional predictive information beyond the baseline clinical model.

Therefore, we used the INSPIRE perioperative database to systematically evaluate the associations and dose–response relationships of geriatric nutritional risk index (GNRI), prognostic nutritional index (PNI), the hemoglobin, albumin, lymphocyte, and platelet (HALP) score, leukocyte-to-albumin ratio (LAR), systemic immune-inflammation index (SII), and advanced lung cancer inflammation index (ALI) with postoperative ICU admission among patients undergoing surgery for hepatobiliary and pancreatic tumors. The incremental predictive performance of these six indices was assessed based on a unified clinical baseline model. We additionally compared GNRI with its individual components and assessed whether the other five indices provided additional information beyond GNRI.

## 2 Methods

### 2.1 Data source and study design

The data for this study were obtained from the INSPIRE perioperative database (30). INSPIRE is a publicly accessible perioperative research database that contains clinical data from patients who underwent surgery and anesthesia management at Seoul National University Hospital between January 2011 and December 2020, covering demographics, diagnosis information, surgical and anesthesia records, laboratory tests, vital signs, in-hospital treatment, and postoperative outcomes. The database was anonymized before public release and approved by the Institutional Review Board of Seoul National University Hospital. Because the data are derived from retrospective clinical records, the requirement for informed consent in the original database study was waived. This study obtained and analyzed de-identified data in accordance with the INSPIRE data use agreement.

### 2.2 Study population

Patients were selected from the INSPIRE database according to predefined criteria (Figure 1). The inclusion criteria were as follows: (1) age ≥18 years; (2) diagnosis of a hepatobiliary or pancreatic tumor; (3) receipt of a qualifying hepatobiliary or pancreatic operation; (4) availability of complete surgical time information, including both operation start and end times, permitting definition of preoperative windows and calculation of operative duration; (5) selection of the earliest qualifying operation recorded in the database for each patient as the index operation; (6) availability of postoperative ICU admission outcome information; and (7) complete data for all six primary nutritional and inflammatory indices. The index operation represented the earliest qualifying operation recorded in the database and did not necessarily correspond to the first operation in the patient’s lifetime.

**Figure 1:**
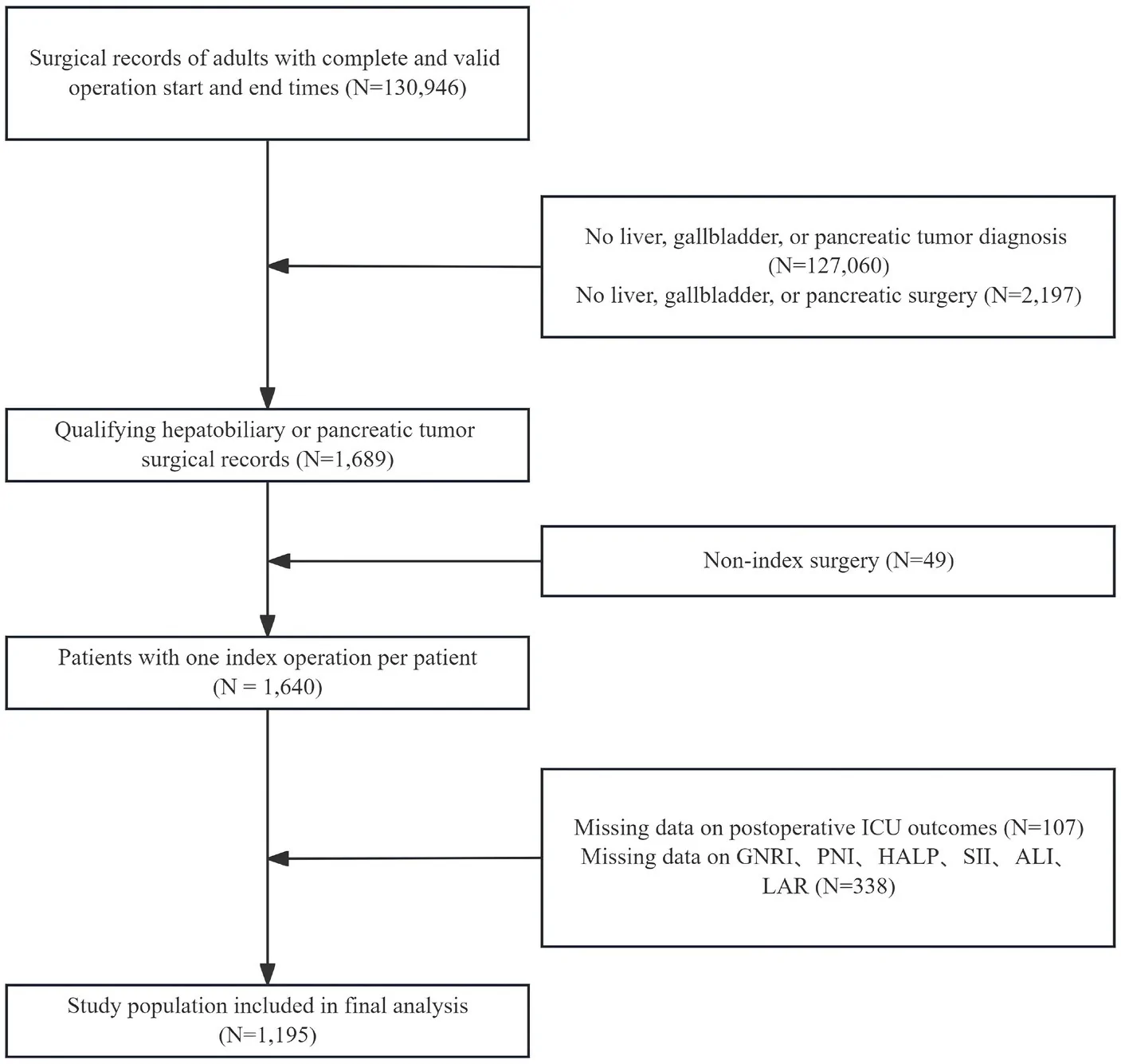
Flowchart of patient selection and study population. GNRI, geriatric nutritional risk index; PNI, prognostic nutritional index; HALP, hemoglobin-albumin-lymphocyte-platelet score; SII, systemic immune-inflammation index; ALI, advanced lung cancer inflammation index; LAR, leukocyte-to-albumin ratio.

### 2.3 Preoperative nutritional and inflammatory indices

This study evaluated GNRI (31), the PNI (32), and the HALP (33). We also evaluated the LAR (34), SII (35), and ALI (36). Higher GNRI, PNI, HALP, and ALI generally indicate a more favorable profile, whereas higher LAR and SII indicate a less favorable profile. All indices were calculated from routinely available anthropometric and laboratory measurements before the index surgery. Measurements within 7 days before surgery were eligible, and the value closest to surgery was selected when multiple measurements were available.

The formulas for each indicator are as follows:

GNRI=1.489×albumin(g/L)+41.7×ABW/IBW

When actual body weight exceeded ideal body weight, the ratio of actual body weight to ideal body weight was set to 1.

$$\mathrm{PNI}=\text{albumin}\;(g/L)+5\times \mathrm{ALC}\;(10^{9}/L)$$

$$\begin{array}{l} \text{HALP}=\text{hemoglobin}\;(g/L)\times \text{albumin}\;(g/L)\times \\ \mathrm{ALC}\;(10^{9}/L)/\mathrm{PLT}\;(10^{9}/L) \end{array}$$

$$\mathrm{LAR}=\mathrm{WBC}\;(10^{9}/L)/\text{albumin}\;(g/L)$$

$$\mathrm{SII}=\mathrm{PLT}\;(10^{9}/L)\times \mathrm{ANC}\;(10^{9}/L)/\mathrm{ALC}\;(10^{9}/L)$$

$$\mathrm{ALI}=\mathrm{BMI}\;(\mathrm{kg}/m^{2})\times \text{albumin}\;(g/\mathrm{dL})/\mathrm{NLR}$$

ABW denotes actual body weight and IBW ideal body weight. ALC, ANC, PLT, and WBC denote absolute lymphocyte, absolute neutrophil, platelet, and white blood cell counts, respectively. BMI denotes body mass index. The neutrophil-to-lymphocyte ratio (NLR) was defined as ANC divided by ALC.

### 2.4 Covariates

The covariates included in this study comprised demographic characteristics, preoperative clinical status, surgery-related factors, preoperative laboratory measurements, and comorbidities. Demographic variables included age, sex, and body mass index (BMI). Preoperative clinical status was assessed using the American Society of Anesthesiologists (ASA) physical status classification, which was categorized into ASA class 1–2 and ≥3. Surgical procedures were classified according to the primary surgical site as liver, gallbladder, or pancreatic surgery. Preoperative laboratory measurements included serum creatinine levels. Comorbidities included chronic liver disease, diabetes, hypertension, and major cardiovascular disease. Major cardiovascular disease was defined as the presence of any of the following conditions before surgery: ischemic heart disease, arrhythmia, or heart failure.

Additional sensitivity variables were operative duration in hours and preoperative alanine aminotransferase (ALT), aspartate aminotransferase (AST), and total bilirubin. Liver biochemistry used the first available measurements within 3 days before surgery. Estimated glomerular filtration rate (eGFR) was calculated using the 2021 race-free CKD-EPI creatinine equation, incorporating age and sex (37). In each imputed dataset, eGFR was recalculated from the completed creatinine value.

### 2.5 Outcome

The primary outcome of this study was postoperative intensive care unit (ICU) admission, determined from the postoperative ICU transfer information recorded in the database. Based on this outcome, patients were divided into a postoperative ICU admission group and a no ICU admission group. The available outcome did not distinguish planned from unplanned ICU admission.

### 2.6 Statistical analysis

Continuous variables were expressed as mean ± standard deviation or median [interquartile range], with the same summary convention across groups for each variable. Categorical variables were presented as frequencies and percentages. Baseline characteristics were described overall and by GNRI category. Clinical characteristics were presented in Table 1, and laboratory measurements and composite indices in Supplementary Table S1.

**Table 1:** Baseline characteristics of patients according to preoperative GNRI categories.

Variable	Overall (*n* = 1,195)	GNRI < 82 (*n* = 27)	82 ≤ GNRI <98 (*n* = 307)	GNRI ≥ 98 (*n* = 861)	*p-*value
Age, years	61.74 ± 11.21	68.70 ± 8.94	64.33 ± 10.93	60.59 ± 11.15	<0.001
BMI, kg/m^2^	23.64 ± 3.39	18.41 ± 2.77	21.86 ± 3.36	24.44 ± 3.01	<0.001
Operative duration, hours	3.08 [2.33–4.08]	2.92 [2.04–4.21]	3.42 [2.42–4.50]	3.00 [2.33–3.92]	<0.001
Sex					0.352
Male	825 (69.0%)	22 (81.5%)	209 (68.1%)	594 (69.0%)	
Female	370 (31.0%)	5 (18.5%)	98 (31.9%)	267 (31.0%)	
ASA group					0.002
ASA 1–2	1,107 (92.6%)	19 (70.4%)	284 (92.5%)	804 (93.4%)	
ASA ≥ 3	88 (7.4%)	8 (29.6%)	23 (7.5%)	57 (6.6%)	
Procedure category					<0.001
Liver	451 (37.7%)	4 (14.8%)	95 (30.9%)	352 (40.9%)	
Gallbladder	587 (49.1%)	19 (70.4%)	179 (58.3%)	389 (45.2%)	
Pancreas	157 (13.1%)	4 (14.8%)	33 (10.7%)	120 (13.9%)	
Chronic liver disease					0.004
No	865 (72.4%)	24 (88.9%)	239 (77.9%)	602 (69.9%)	
Yes	330 (27.6%)	3 (11.1%)	68 (22.1%)	259 (30.1%)	
Diabetes					0.373
No	1,086 (90.9%)	23 (85.2%)	283 (92.2%)	780 (90.6%)	
Yes	109 (9.1%)	4 (14.8%)	24 (7.8%)	81 (9.4%)	
Hypertension					0.233
No	1,107 (92.6%)	24 (88.9%)	290 (94.5%)	793 (92.1%)	
Yes	88 (7.4%)	3 (11.1%)	17 (5.5%)	68 (7.9%)	
Major cardiovascular disease					0.772
No	1,165 (97.5%)	27 (100.0%)	301 (98.0%)	837 (97.2%)	
Yes	30 (2.5%)	0 (0.0%)	6 (2.0%)	24 (2.8%)	

Continuous variables are presented as mean ± standard deviation (SD) or median [interquartile range (IQR)], as appropriate, and categorical variables as number (percentage). GNRI was categorized using prespecified clinical cutoffs as <82, 82 to <98, and ≥98. BMI, body mass index; GNRI, geriatric nutritional risk index; ASA, American Society of Anesthesiologists; SD, standard deviation; IQR, interquartile range.

Missing data were handled using multiple imputation by chained equations with the R package mice (38). The imputation model included the outcome, all six primary indices, clinical covariates, operative duration, and liver biochemistry. Predictive mean matching was used for ASA score, creatinine, ALT, AST, and total bilirubin. Multinomial logistic regression was specified for the multicategory procedure variable. Twenty datasets were generated using 20 iterations and seed 2026. The observed outcome predicted other variables but was not imputed. Missingness before imputation is reported in Supplementary Table S2. eGFR and transformed variables were derived after imputation. Logistic models were fitted in every completed dataset, and log-odds coefficients and their variances were pooled using Rubin’s rules (39). Odds ratios (ORs) and 95% confidence intervals (CIs) were obtained by exponentiating the pooled estimates and confidence limits.

Logistic regression models were used to evaluate the associations of GNRI, PNI, HALP, LAR, SII, and ALI with postoperative ICU admission. Because the six indicators differed in distributional characteristics and numerical scale, each indicator underwent log1p transformation before being entered into the regression model as a continuous variable, followed by *Z*-score standardization. In the continuous-variable models, the odds ratio (OR) and its 95% confidence interval (CI) represent the odds of postoperative ICU admission associated with each 1-standard-deviation increase in the transformed and standardized indicator. Three logistic regression models were constructed: a non-adjusted model with no covariate adjustment; Adjusted Model I, which adjusted for age, sex, and body mass index; and Adjusted Model II, which further adjusted for ASA class, surgery type, preoperative serum creatinine, chronic liver disease, diabetes, hypertension, and major cardiovascular disease.

In categorical analysis, GNRI was categorized as <82, 82 to <98, and ≥98 using external cut points (31), with <82 as the reference. These thresholds were retained for continuity with published nutritional risk classifications, not assumed to be optimal for this surgical cohort. PNI, HALP, LAR, SII, and ALI used study-population tertiles, with T1 as the reference. Trend tests modeled the within-category median index value as a continuous variable. A sensitivity analysis also categorized GNRI using study-population tertiles.

Restricted cubic spline (RCS) models evaluated dose–response relationships after adjustment for the covariates in Adjusted II. Log-OR contrasts were pooled using Rubin’s rules. Overall and nonlinear associations were tested using pooled D1 joint Wald comparisons of spline, linear, and covariate-only models.

Prespecified subgroup analyses were performed to assess whether the associations between the six indices, modeled as continuous variables per 1-standard-deviation increase, and postoperative ICU admission varied across age (<65 vs. ≥65 years), sex, BMI (<24 vs. ≥24 kg/m^2^), ASA group (1–2 vs. ≥3), procedure category (liver, gallbladder, or pancreas), chronic liver disease, diabetes, hypertension, and major cardiovascular disease. Within each subgroup, models adjusted for Adjusted II covariates except the subgroup-defining variable. Interaction terms were tested using Rubin-pooled Wald tests for single coefficients or D1 joint Wald tests for multiple coefficients. Benjamini–Hochberg false discovery rate (FDR) correction was applied across all interaction tests. Subgroup analyses were interpreted as exploratory. Sparse strata without stable estimates were reported as not estimable.

To compare incremental performance, each continuous index was added separately to the clinical model defined by the Adjusted II covariates. Discrimination was assessed using AUC, and prediction error using the Brier score. ΔAUC was the extended-model AUC minus the clinical-model AUC. Brier-score improvement was the clinical-model score minus the extended-model score. Each metric and model contrast was calculated within each completed dataset, then averaged arithmetically across the 20 datasets. Calibration and decision curves were averaged from within-imputation estimates.

Continuous net reclassification improvement (NRI) and integrated discrimination improvement (IDI) compared extended and reference model risks in the same participants. No prespecified risk categories were used. Continuous NRI favored upward risk changes among events and downward changes among nonevents. IDI was the change in the event-versus-nonevent mean risk difference. NRI and IDI were calculated within each completed dataset and averaged across imputations. For performance uncertainty, 1,000 bootstrap samples were drawn with replacement from the original incomplete cohort. Each sample underwent fresh MICE with 20 datasets and model refitting before metric averaging. The same sampled participants were used for paired model comparisons. Percentile 95% CIs used the 2.5th and 97.5th percentiles of the bootstrap estimates.

GNRI component models added albumin, the capped actual-to-ideal weight ratio, both components, or GNRI to the same clinical reference model. Separate models then added PNI, HALP, SII, ALI, or LAR to the clinical model plus GNRI. The joint component model estimated separate coefficients and was not equivalent to the fixed-weight GNRI composite. These comparisons used the same within-imputation averaging and paired bootstrap-then-imputation procedure.

Bootstrap internal validation estimated optimism for the clinical model and six index-extended models. Within the 20 completed datasets, 1,000 shared participant resamples were used to refit each model. Optimism was the bootstrap-sample AUC minus the AUC obtained by applying that fitted model to the corresponding completed cohort. Mean optimism across resamples and imputations was subtracted from mean apparent AUC. The mean bootstrap validation calibration slope estimated potential global shrinkage. Corrected-AUC 95% CIs used 500 outer resamples of the original incomplete cohort, fresh MICE with 20 datasets, and 100 inner resamples per outer sample. Percentile limits were calculated from the resulting corrected AUCs. This was internal validation, not external validation.

As a sensitivity analysis, we repeated the logistic regression and incremental predictive analyses using a complete-case cohort derived from the original dataset before multiple imputation. Participants were retained only if they had complete data for postoperative ICU admission, all six nutritional and inflammatory indices, and all covariates included in Adjusted Model II. This yielded 1,146 participants, including 130 postoperative ICU admissions. The three logistic regression models were refitted in this common complete-case cohort, and the continuous, tertile, and trend analyses were repeated. Incremental predictive performance was re-evaluated by comparing the clinical baseline model with each extended model using the AUC, ΔAUC, Brier score, NRI, IDI, calibration analysis, and decision curve analysis.

Additional logistic sensitivity analyses were performed separately. Adjusted III added log1p-transformed ALT, AST, and total bilirubin to Adjusted II. Adjusted IV added operative duration in hours to Adjusted II, without the liver variables. Adjusted V replaced creatinine with eGFR while retaining the other Adjusted II covariates. These models were fitted across all 20 completed datasets and Rubin-pooled. A separate complete-case analysis recalculated all six indices from the first available laboratory measurements within 3 days before surgery. It included 1,040 participants and 114 ICU admissions and repeated continuous, categorical, and median-score trend analyses.

All tests were two-sided, and *p* < 0.05 was considered statistically significant. All statistical analyses were performed using R software.

## 3 Results

### 3.1 Baseline characteristics

A total of 1,195 patients were included, of whom 141 (11.8%) experienced postoperative ICU admission. The mean age was 61.74 ± 11.21 years, 69.0% were male, and 7.4% had ASA class ≥3. Liver, gallbladder, and pancreatic procedures accounted for 37.7%, 49.1%, and 13.1%, respectively. Median operative duration was 3.08 h. GNRI groups <82, 82 to <98, and ≥98 included 27, 307, and 861 patients. The lowest GNRI group was older and had lower BMI than the highest group (68.70 versus 60.59 years, 18.41 versus 24.44 kg/m^2^, both *p* < 0.001). ASA class ≥3 was also more frequent (29.6% versus 6.6%) (Table 1). Laboratory characteristics are reported separately in Supplementary Table S1. Before imputation, creatinine was missing in 24 patients (2.01%) and ASA score in 18 (1.51%). ALT and AST were each missing in 104 patients (8.70%), and total bilirubin in 106 (8.87%) (Supplementary Table S2).

### 3.2 Associations of nutritional and inflammatory indices with postoperative ICU admission

Logistic regression analyses showed that all six indices were associated with postoperative ICU admission (Table 2). In the fully adjusted model (Adjusted II), each 1-standard deviation increase in GNRI was associated with a lower probability of postoperative ICU admission (OR 0.601; 95% CI, 0.497–0.728). Similar inverse associations were observed for PNI (OR 0.650; 95% CI, 0.541–0.780), HALP (OR 0.664; 95% CI, 0.550–0.801), and ALI (OR 0.674; 95% CI, 0.559–0.812). Higher LAR (OR 1.434; 95% CI, 1.209–1.701) and SII (OR 1.430; 95% CI, 1.192–1.716) were associated with an increased probability of postoperative ICU admission.

**Table 2:** Logistic regression analyses of the associations between nutritional and inflammatory indices and postoperative ICU admission.

Exposures	Non-adjusted	Adjusted I	Adjusted II
GNRI tertiles			
GNRI (per SD change)	0.581 (0.499–0.676)^***^	0.565 (0.471–0.677)^***^	0.601 (0.497–0.728)^***^
T1	1 (ref)	1 (ref)	1 (ref)
T2	0.422 (0.184–0.967)^*^	0.421 (0.174–1.018)	0.538 (0.213–1.356)
T3	0.150 (0.066–0.341)^***^	0.152 (0.060–0.382)^***^	0.209 (0.080–0.548)^**^
*p* for trend	<0.001	<0.001	<0.001
ALI tertiles			
ALI (per SD change)	0.589 (0.503–0.691)^***^	0.621 (0.519–0.744)^***^	0.674 (0.559–0.812)^***^
T1	1 (ref)	1 (ref)	1 (ref)
T2	0.409 (0.266–0.626)^***^	0.445 (0.285–0.693)^***^	0.487 (0.306–0.774)^**^
T3	0.344 (0.220–0.539)^***^	0.417 (0.254–0.684)^**^	0.471 (0.280–0.791)^**^
*p* for trend	<0.001	<0.001	0.003
HALP tertiles			
HALP (per SD change)	0.576 (0.488–0.679)^***^	0.606 (0.506–0.726)^***^	0.664 (0.550–0.801)^***^
T1	1 (ref)	1 (ref)	1 (ref)
T2	0.383 (0.251–0.586)^***^	0.424 (0.272–0.660)^***^	0.469 (0.295–0.748)^**^
T3	0.277 (0.174–0.442)^***^	0.310 (0.188–0.508)^***^	0.379 (0.226–0.636)^***^
*p* for trend	<0.001	<0.001	<0.001
LAR tertiles			
LAR (per SD change)	1.671 (1.428–1.956)^***^	1.539 (1.308–1.812)^***^	1.434 (1.209–1.701)^***^
T1	1 (ref)	1 (ref)	1 (ref)
T2	1.436 (0.865–2.383)	1.247 (0.744–2.090)	1.146 (0.672–1.953)
T3	3.009 (1.900–4.765)^***^	2.406 (1.501–3.858)^***^	2.060 (1.263–3.361)^**^
*p* for trend	<0.001	<0.001	0.001
PNI tertiles			
PNI (per SD change)	0.577 (0.491–0.678)^***^	0.618 (0.519–0.735)^***^	0.650 (0.541–0.780)^***^
T1	1 (ref)	1 (ref)	1 (ref)
T2	0.549 (0.368–0.818)^**^	0.612 (0.405–0.923)^*^	0.628 (0.408–0.965)^*^
T3	0.239 (0.144–0.397)^***^	0.297 (0.176–0.500)^***^	0.335 (0.195–0.576)^***^
*p* for trend	<0.001	<0.001	<0.001
SII tertiles			
SII (per SD change)	1.711 (1.448–2.022)^***^	1.562 (1.311–1.861)^***^	1.430 (1.192–1.716)^***^
T1	1 (ref)	1 (ref)	1 (ref)
T2	1.510 (0.898–2.539)	1.422 (0.840–2.406)	1.312 (0.762–2.260)
T3	3.421 (2.141–5.468)^***^	2.793 (1.723–4.528)^***^	2.393 (1.448–3.954)^**^
*p* for trend	<0.001	<0.001	<0.001

Continuous indices were analyzed per standard deviation increase after log1p transformation and *Z*-score standardization. GNRI categories were <82 (reference), 82 to <98, and ≥98. The other five indices used study-population tertiles, with T1 as reference. The non-adjusted model included no covariates. Adjusted I included age, sex, and BMI. Adjusted II additionally included ASA group, procedure category, creatinine, chronic liver disease, diabetes, hypertension, and major cardiovascular disease. ORs and 95% CIs were pooled across 20 completed datasets using Rubin’s rules. ^*^p < 0.05, ^**^p < 0.01, ^***^p < 0.001. BMI, body mass index; ASA, American Society of Anesthesiologists; OR, odds ratio; CI, confidence interval; GNRI, geriatric nutritional risk index; PNI, prognostic nutritional index; HALP, hemoglobin, albumin, lymphocyte, and platelet score; LAR, leukocyte-to-albumin ratio; SII, systemic immune-inflammation index; ALI, advanced lung cancer inflammation index; ICU, intensive care unit.

Categorical analyses showed consistent graded associations. In the Adjusted II model, compared with the reference category, GNRI ≥98 versus <82 was associated with lower odds of postoperative ICU admission (OR 0.209; 95% CI, 0.080–0.548). Compared with the lowest tertile, the highest tertiles of PNI (OR 0.335; 95% CI, 0.195–0.576), HALP (OR 0.379; 95% CI, 0.226–0.636), and ALI (OR 0.471; 95% CI, 0.280–0.791) were associated with lower odds of postoperative ICU admission, whereas the highest tertiles of LAR (OR 2.060; 95% CI, 1.263–3.361) and SII (OR 2.393; 95% CI, 1.448–3.954) were associated with higher odds. Trend tests across categories were statistically significant for all six indices (all *p* for trend ≤0.003).

In the sensitivity analysis, the results from the continuous-variable analyses were consistent with those of the main analysis. In the Adjusted II model, the ORs per 1-standard-deviation increase in GNRI, PNI, HALP, and ALI were 0.576 (95% CI, 0.473–0.701), 0.646 (95% CI, 0.535–0.780), 0.663 (95% CI, 0.546–0.804), and 0.665 (95% CI, 0.547–0.808), respectively. The ORs for LAR and SII were 1.487 (95% CI, 1.246–1.776) and 1.453 (95% CI, 1.202–1.756), respectively. Trend tests were statistically significant for all six indices (Supplementary Table S3).

GNRI also showed an inverse association when categorized by study-population tertiles (T3 versus T1 OR 0.309; 95% CI, 0.179–0.532) (Supplementary Table S4). Its continuous OR was attenuated to 0.678 (0.556–0.828) after liver-biochemistry adjustment and 0.667 (0.530–0.839) after operative-duration adjustment. Replacing creatinine with eGFR yielded an OR of 0.599 (0.495–0.725) (Supplementary Table S5). All six continuous associations retained their primary directions, although some categorical trends were attenuated after operative-duration adjustment. In the 3-day complete-case analysis, the GNRI OR was 0.599 (0.488–0.737), with consistent directions for the other indices (Supplementary Table S6).

### 3.3 Subgroup analyses

Subgroup estimates are shown in Supplementary Figures S1–S6. The interactions were observed for GNRI with age (*p* = 0.014) and chronic liver disease (*p* = 0.032), and for LAR with chronic liver disease (*p* = 0.023). None of the 54 interactions met the FDR threshold of 0.05. These three adjusted *p* values were each 0.543 (Supplementary Table S7). These findings were therefore treated as exploratory rather than evidence of established subgroup differences.

### 3.4 Dose–response associations with postoperative ICU admission

RCS analysis showed that after multivariable adjustment, all six indicators had an overall association with postoperative ICU admission (Figure 2). As the levels of GNRI, ALI, HALP, and PNI increased, the adjusted odds of postoperative ICU admission decreased gradually. As the SII level increased, the adjusted odds of ICU admission increased gradually. LAR also showed a positive association with ICU admission in the higher range. However, no significant nonlinear relationship was observed for any of the six indicators (all *p* for nonlinear > 0.05).

**Figure 2:**
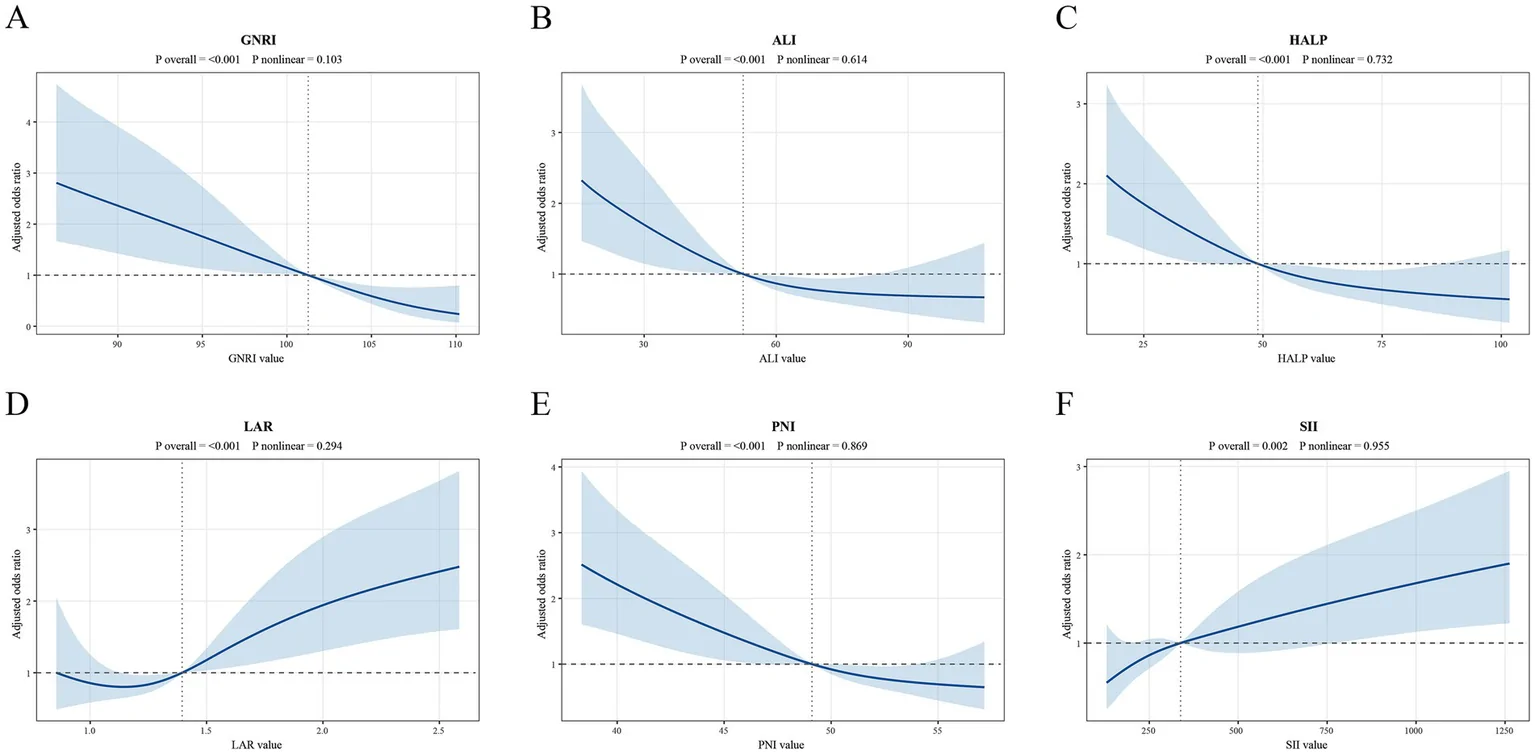
RCS analysis of the associations between nutritional and inflammatory indices and postoperative ICU admission. **(A)** GNRI, **(B)** ALI, **(C)** HALP, **(D)** LAR, **(E)** PNI, **(F)** SII. Models were adjusted for age, sex, BMI, ASA group, procedure category, creatinine, chronic liver disease, diabetes, hypertension, and major cardiovascular disease. Overall and nonlinear *p* values use MI-pooled D1 joint Wald tests. GNRI, geriatric nutritional risk index; ALI, advanced lung cancer inflammation index; HALP, hemoglobin-albumin-lymphocyte-platelet score; LAR, leukocyte-to-albumin ratio; PNI, prognostic nutritional index; BMI, body mass index; ASA, American Society of Anesthesiologists.

### 3.5 Model predictive performance

The clinical model had an AUC of 0.766 (95% CI, 0.728–0.805). Adding GNRI yielded the highest observed AUC, 0.800 (0.764–0.835). AUCs for PNI, LAR, HALP, ALI, and SII were 0.787, 0.787, 0.786, 0.785, and 0.783, respectively (Figure 3A; Supplementary Table S8). Calibration curves are shown in Figure 3B. The clinical-model Brier score was 0.094 (95% CI, 0.082–0.107), decreasing to 0.092 (0.081–0.104) with GNRI or PNI. Scores were approximately 0.093 after adding the other indices. Decision curves suggested potential net benefit over parts of the evaluated threshold range (Figure 3C).

**Figure 3:**
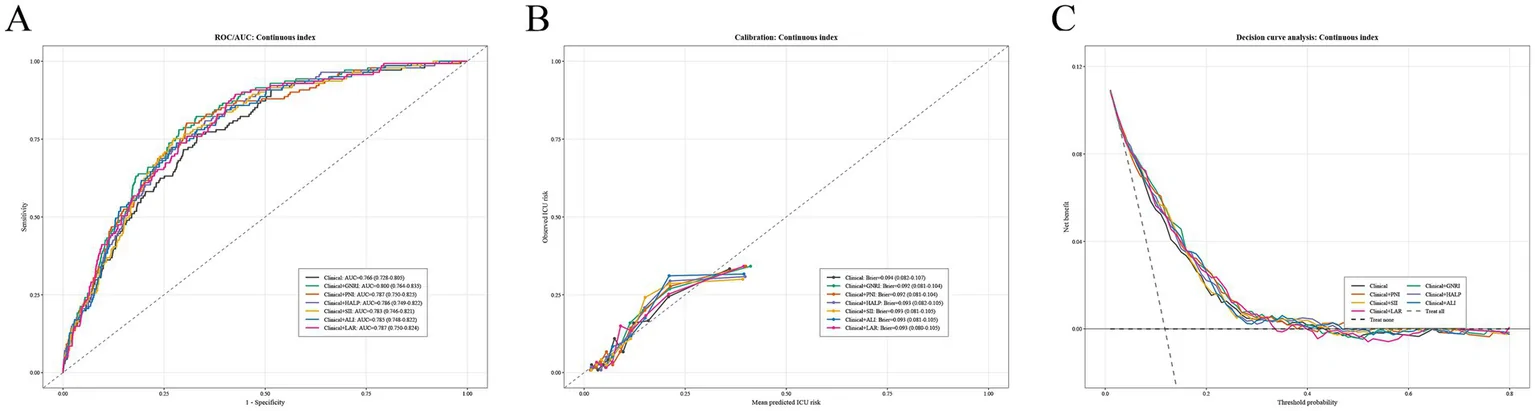
Discrimination, calibration, and clinical utility of the clinical model and models that separately added each continuous nutritional or inflammatory index for postoperative ICU admission. **(A)** *AUC* analysis, **(B)** calibration analysis, **(C)** decision curve analysis. *AUC* and Brier score point estimates are arithmetic means across the 20 imputed datasets. 95% *CIs* were derived from 1,000 multiple-imputation-aware bootstrap resamples of the original incomplete cohort, with *MICE* repeated and the models refitted in each resample. Calibration and *DCA* curves show mean within-imputation estimates. AUC, area under the receiver operating characteristic curve; DCA, decision curve analysis; GNRI, geriatric nutritional risk index; PNI, prognostic nutritional index; HALP, hemoglobin-albumin-lymphocyte-platelet score; SII, systemic immune-inflammation index; ALI, advanced lung cancer inflammation index; LAR, leukocyte-to-albumin ratio; ICU, intensive care unit.

### 3.6 Incremental predictive performance

GNRI produced the largest observed ΔAUC of 0.033. Corresponding increments were 0.021 for PNI and LAR, 0.020 for HALP, 0.018 for ALI, and 0.017 for SII (Figure 4A; Supplementary Table S8). Differences were calculated before rounding the model AUCs. GNRI and PNI showed the largest Brier-score reductions (Figure 4B). After adding GNRI to the clinical baseline model, the continuous NRI was 0.312 (95% CI, 0.139–0.487), and IDI was 0.028 (95% CI, 0.013–0.045). GNRI had the largest observed IDI, whereas ALI had the largest continuous NRI (0.355, 95% CI, 0.185–0.520). Other index estimates are reported in Supplementary Table S9.

**Figure 4:**
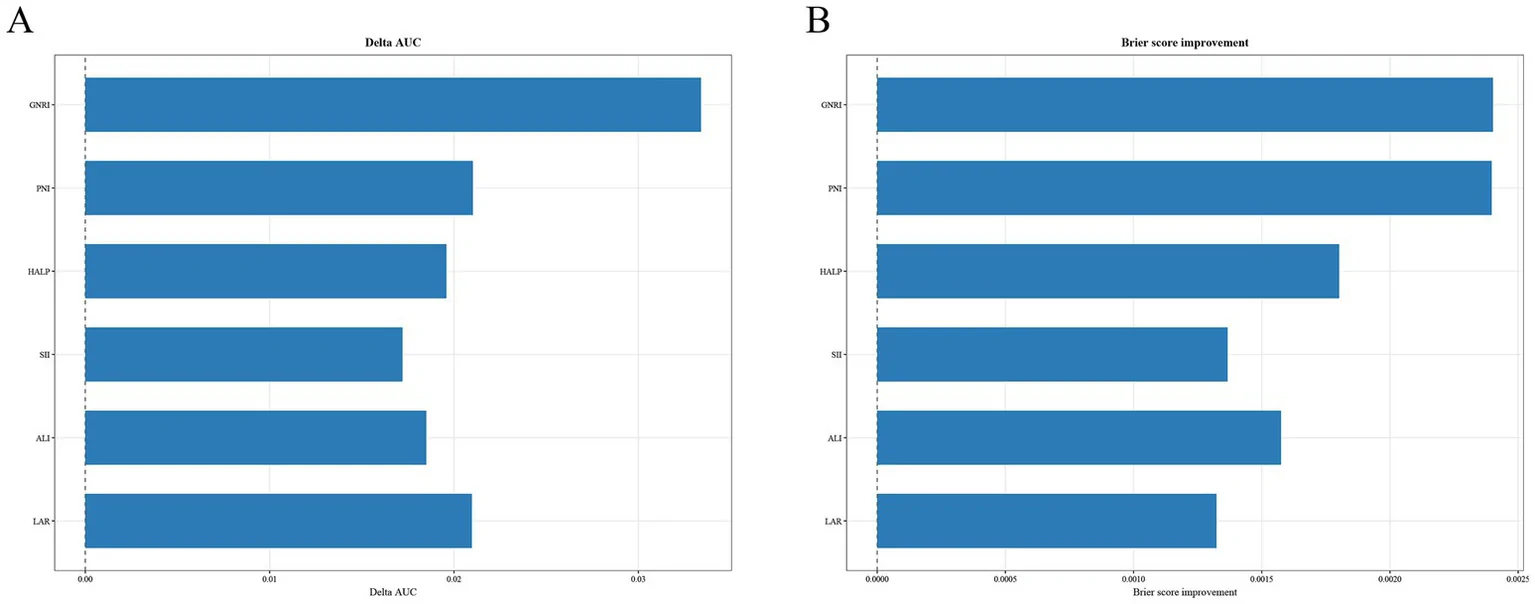
Incremental predictive performance for postoperative ICU admission after each continuous nutritional or inflammatory index was added separately to the clinical model. **(A)** *ΔAUC*. **(B)** Brier score improvement. Bars show point estimates averaged across the 20 imputed datasets. *ΔAUC* is extended-model *AUC* minus clinical-model *AUC*. Brier-score improvement is clinical-model score minus extended-model score. Positive values indicate improvement. *ΔAUC*, change in *AUC*; *AUC*, area under the receiver operating characteristic curve; GNRI, geriatric nutritional risk index; PNI, prognostic nutritional index; HALP, hemoglobin-albumin-lymphocyte-platelet score; SII, systemic immune-inflammation index; ALI, advanced lung cancer inflammation index; LAR, leukocyte-to-albumin ratio; ICU, intensive care unit.

In the sensitivity analysis, the clinical baseline model had an AUC of 0.769 (95% CI, 0.729–0.810). After adding GNRI, the AUC increased to 0.803 (95% CI, 0.765–0.840), corresponding to a ΔAUC of 0.033, the highest among the six indicators. The IDI for GNRI was also the highest, at 0.035 (95% CI, 0.017–0.055) (Supplementary Tables S10–S11; Supplementary Figures S7, S8).

After internal validation, optimism-corrected AUC was 0.744 (95% CI, 0.707–0.783) for the clinical model and 0.780 (0.744–0.816) with GNRI. Mean optimism for the GNRI model was 0.020, and its mean bootstrap validation calibration slope was 0.897. Corrected AUCs for the other extended models ranged from 0.761 to 0.767 (Supplementary Table S12). Albumin alone and albumin plus weight ratio each yielded an AUC of 0.799, compared with 0.800 for GNRI. Weight ratio alone yielded 0.768 (Supplementary Table S13). Reclassification estimates for these models are reported in Supplementary Table S14. Adding another index to the GNRI model changed AUC by −0.001 to 0.006 (Supplementary Table S15). All corresponding continuous NRI intervals included zero (Supplementary Table S16), indicating uncertain additional reclassification gains.

## 4 Discussion

This study compared six preoperative nutritional and inflammatory indices in 1,195 patients undergoing hepatobiliary or pancreatic tumor surgery. Higher GNRI, PNI, HALP, and ALI were associated with lower odds of postoperative ICU admission, whereas higher LAR and SII showed positive associations. These associations remained consistent after multivariable adjustment and in sensitivity analyses. GNRI had the highest observed incremental AUC and IDI, with a numerical discrimination advantage that persisted after internal optimism correction. However, albumin alone performed similarly, and other indices added limited discrimination beyond GNRI.

The consistent risk direction observed across the six indicators reflects the importance of preoperative nutritional reserve and systemic inflammatory status in patients undergoing hepatobiliary or pancreatic tumor surgery. GNRI, PNI, HALP, LAR, and ALI all incorporate albumin to varying degrees. Low albumin not only reflects decreased nutritional reserve but is also associated with inflammation-mediated increased capillary permeability, albumin redistribution, and metabolic alterations (40). In patients with hepatobiliary or pancreatic tumors, biliary obstruction (41), impaired hepatic synthetic function (42), and pancreatic exocrine dysfunction (43) can further exacerbate the decline in protein and energy reserves. At the same time, elevated neutrophil counts, lymphopenia, and platelet activation reflect systemic inflammation and immune imbalance, changes that have been associated with postoperative infection, organ dysfunction, and poor recovery (44). The hemoglobin component of HALP further reflects tissue oxygen delivery capacity, whereas preoperative anemia can reduce the patient’s ability to compensate for surgical blood loss and the increased perioperative oxygen demand (45). Therefore, lower GNRI, PNI, HALP, and ALI, along with higher LAR and SII, may collectively reflect reduced physiological reserve and a greater inflammatory burden, thereby increasing the need for intensive monitoring and organ support after major hepatobiliary or pancreatic surgery.

Among the six evaluated indices, GNRI showed the largest observed incremental improvement in discrimination. Adding GNRI to the clinical baseline model increased the apparent AUC from 0.766 to 0.800, and GNRI also showed the largest observed IDI. Its numerical advantage in discrimination persisted after internal optimism correction. These findings suggest that GNRI may serve as an informative composite marker for preoperative risk assessment in patients undergoing hepatobiliary or pancreatic tumor surgery.

However, further component analysis showed that a substantial proportion of the discriminatory information captured by GNRI overlapped with that provided by albumin. Albumin alone yielded an apparent AUC of 0.799, compared with 0.800 for GNRI. In contrast, the actual-to-ideal body weight ratio alone yielded an AUC of 0.768. Adding the weight ratio to albumin did not materially improve discrimination. Therefore, although GNRI integrates albumin and weight-related information in a composite form, the present data do not establish an advantage of GNRI over albumin alone. Albumin reflects not only nutritional status but also inflammatory and hepatic processes (40–43), which may partly explain its strong predictive contribution. The attenuation of the association between GNRI and the outcome after further adjustment for liver biochemistry also suggests some overlap in the clinical information captured by these variables. However, ALT, AST, and bilirubin do not fully characterize hepatic synthetic reserve. In models already including GNRI, adding any of the other five indices changed the AUC by only −0.001 to 0.006, and the corresponding reclassification estimates remained uncertain. Overall, these findings support the prognostic relevance of GNRI while also indicating that the incremental value of overlapping composite scores should be compared with that of simpler, routinely available markers before their use in clinical risk assessment.

Nominal subgroup interactions were observed for GNRI with age and chronic liver disease and for LAR with chronic liver disease. However, none remained significant after FDR correction. Therefore, the present data do not establish that the associations of these indices with postoperative ICU admission differ across age or chronic liver disease subgroups, and the observed subgroup patterns should be considered exploratory. GNRI was originally developed for nutritional risk assessment in older adults, and its conventional cut points should not be assumed to be optimal for younger surgical populations. The association of GNRI with postoperative ICU admission remained consistent when GNRI was categorized using study-population tertiles, supporting the robustness of the observed association to alternative categorization. However, further studies are needed to determine whether a clinically useful GNRI cutoff can be defined for perioperative risk assessment.

This study has several limitations. First, INSPIRE is a single-center retrospective database, which may limit the generalizability of the findings to other institutions, patient populations, and perioperative care settings. ICU admission practices, monitoring strategies, and resource availability may differ across centers. Although bootstrap internal validation was performed to quantify optimism, the present estimates remain specific to this cohort. Second, the available outcome did not distinguish planned from unplanned postoperative ICU admission. Consequently, ICU admission may reflect institutional monitoring practices and resource allocation as well as the severity of postoperative illness. This limits interpretation of ICU admission as a direct surrogate for postoperative complications or physiological deterioration. Third, residual confounding cannot be excluded. Important clinical factors, including tumor stage, functional status, frailty, sarcopenia, and preoperative nutritional interventions, were not available in the analysis dataset. Detailed surgical extent was also not reliably captured beyond organ-level procedure categories, and transfusion records were too sparse for stable adjustment. These unmeasured or incompletely measured factors may influence both preoperative nutritional and inflammatory indices and the likelihood of postoperative ICU admission. Fourth, operative duration was available only after surgery and therefore could not be considered a true preoperative predictor. It was included only in sensitivity analyses to examine whether the observed associations were partly explained by operative complexity. Similarly, adjustment for ALT, AST, and total bilirubin provided only a partial assessment of liver-related clinical information because these markers do not fully characterize hepatic synthetic reserve or the severity of chronic liver disease. Fifth, creatinine-based eGFR, although calculated using the 2021 race-free CKD-EPI equation and incorporating age and sex, remains influenced by muscle mass. Because age and sex were already included in the primary model, replacing creatinine with eGFR does not address unmeasured sarcopenia or other body-composition differences. In addition, the nutritional and inflammatory indices were derived from routinely available measurements obtained within predefined preoperative windows and may therefore be affected by transient inflammatory, metabolic, or hydration-related changes. Single preoperative measurements also do not capture longitudinal changes in nutritional or inflammatory status. Finally, before these indices are incorporated into perioperative clinical decision-making, external validation in independent cohorts is needed, together with further assessment of calibration across different settings and clinical impact.

## 5 Conclusion

Preoperative nutritional and inflammatory indices were associated with postoperative ICU admission after hepatobiliary and pancreatic tumor surgery. GNRI showed the highest observed incremental discrimination, but albumin alone performed similarly and other indices added limited information beyond GNRI. These routinely available markers warrant further evaluation for preoperative risk assessment. External validation and assessment of clinical impact are needed before using them to guide ICU allocation or nutritional interventions.

## Data Availability

The data analyzed in this study is subject to the following licenses/restrictions: The INSPIRE database contains de-identified perioperative clinical data. Individual-level data are not publicly available for unrestricted download because access is controlled by the INSPIRE data use agreement. Access is limited to qualified researchers who submit a data access request and obtain approval. The data may be used only for the approved research purpose and may not be redistributed or used to attempt re-identification, in accordance with the INSPIRE data use agreement and applicable privacy and ethical requirements. Requests to access these datasets should be directed to PhysioNet INSPIRE Dataset, https://physionet.org/content/inspire/1.4.2/.
