# Supplementary material for: Association and incremental predictive performance of preoperative nutritional and inflammatory indices for postoperative ICU admission after hepatobiliary and pancreatic tumor surgery
Source: Front Nutr. 2026 Oct 22;13:1978034. doi: 10.3389/fnut.2026.1978034 (PMC13736577; doi:10.3389/fnut.2026.1978034)
Supplement: Supplementary file 1 [file Supplementary_file_1.DOCX]

Supplementary Material

# 1 Supplementary Figures and Tables

## 1.1 Supplementary Figures

**Figure S1.** Forest plot of the subgroup analyses of the association between continuous ALI and postoperative ICU admission. Odds ratios (ORs) and 95% confidence intervals (CIs) were estimated across prespecified subgroups. ALI, advanced lung cancer inflammation index; CI, confidence interval; OR, odds ratio.

**Figure S2.** Forest plot of the subgroup analyses of the association between continuous GNRI and postoperative ICU admission. Odds ratios (ORs) and 95% confidence intervals (CIs) were estimated across prespecified subgroups. GNRI, geriatric nutritional risk index; CI, confidence interval; OR, odds ratio.

**Figure S3.** Forest plot of the subgroup analyses of the association between continuous HALP and postoperative ICU admission. Odds ratios (ORs) and 95% confidence intervals (CIs) were estimated across prespecified subgroups. HALP, hemoglobin-albumin-lymphocyte-platelet score; CI, confidence interval; OR, odds ratio; NE, not estimable.

**Figure S4.** Forest plot of the subgroup analyses of the association between continuous LAR and postoperative ICU admission. Odds ratios (ORs) and 95% confidence intervals (CIs) were estimated across prespecified subgroups. LAR, leukocyte-to-albumin ratio; CI, confidence interval; OR, odds ratio; NE, not estimable.

**Figure S5.** Forest plot of the subgroup analyses of the association between continuous PNI and postoperative ICU admission. Odds ratios (ORs) and 95% confidence intervals (CIs) were estimated across prespecified subgroups. PNI, prognostic nutritional index; CI, confidence interval; OR, odds ratio; NE, not estimable.

**Figure S6.** Forest plot of the subgroup analyses of the association between continuous SII and postoperative ICU admission. Odds ratios (ORs) and 95% confidence intervals (CIs) were estimated across prespecified subgroups. SII, systemic immune-inflammation index; CI, confidence interval; OR, odds ratio.

**Figure S7.** Sensitivity analysis of the predictive performance of the clinical model and clinical models incorporating individual continuous nutritional and inflammatory indices in the complete-case cohort. (A) AUC analysis. (B) Calibration analysis. (C) Decision curve analysis. AUC, area under the curve; DCA, decision curve analysis; GNRI, geriatric nutritional risk index; PNI, prognostic nutritional index; HALP, hemoglobin-albumin-lymphocyte-platelet score; SII, systemic immune-inflammation index; ALI, advanced lung cancer inflammation index; LAR, leukocyte-to-albumin ratio.

**Figure S8.** Sensitivity analysis of the incremental predictive performance of the clinical model after adding individual continuous nutritional and inflammatory indices in the complete-case cohort. (A) ΔAUC. (B) Brier score improvement. ΔAUC, change in the area under the curve; GNRI, geriatric nutritional risk index; PNI, prognostic nutritional index; HALP, hemoglobin-albumin-lymphocyte-platelet score; SII, systemic immune-inflammation index; ALI, advanced lung cancer inflammation index; LAR, leukocyte-to-albumin ratio.

## 1.2 Supplementary Tables

**Table S1.** Baseline laboratory characteristics and nutritional and inflammatory indices according to GNRI categories.

| Variable | Overall (n=1,195) | GNRI <82 (n=27) | 82 ≤ GNRI <98 (n=307) | GNRI ≥98 (n=861) | *P* value |
| --- | --- | --- | --- | --- | --- |
| Creatinine, mg/dL | 0.84 [0.71-0.98] | 0.71 [0.61-0.95] | 0.80 [0.68-0.92] | 0.84 [0.71-0.98] | <0.001 |
| Albumin, g/dL | 3.99 ± 0.40 | 2.87 ± 0.30 | 3.61 ± 0.31 | 4.16 ± 0.25 | <0.001 |
| Hemoglobin, g/dL | 13.17 ± 1.63 | 10.61 ± 1.66 | 12.41 ± 1.67 | 13.52 ± 1.45 | <0.001 |
| White blood cell, ×10^9^/L | 5.58 [4.77-6.67] | 7.45 [5.39-10.30] | 5.58 [4.24-7.05] | 5.58 [4.77-6.67] | 0.001 |
| Lymphocytes, % | 31.00 ± 9.25 | 15.82 ± 8.33 | 28.38 ± 10.04 | 32.41 ± 8.32 | <0.001 |
| Platelet, ×10^9^/L | 200.17 ± 74.71 | 280.19 ± 113.06 | 203.28 ± 85.89 | 196.55 ± 67.23 | <0.001 |
| Neutrophils, % | 57.15 ± 9.96 | 72.84 ± 9.69 | 58.97 ± 10.79 | 56.00 ± 9.12 | <0.001 |
| Lymphocyte count, ×10^9^/L | 1.77 ± 0.66 | 1.17 ± 0.62 | 1.61 ± 0.68 | 1.85 ± 0.64 | <0.001 |
| Neutrophil count, ×10^9^/L | 3.12 [2.45-4.07] | 5.05 [4.06-8.32] | 3.12 [2.36-4.37] | 3.12 [2.46-3.84] | <0.001 |
| eGFR, mL/min/1.73m^2^ | 96.12 [85.56-102.99] | 98.26 [83.52-104.98] | 96.31 [86.75-102.49] | 95.68 [83.83-102.99] | 0.755 |
| ALT, U/L | 27.00 [17.00-35.00] | 22.00 [20.00-44.00] | 27.00 [17.00-42.00] | 24.00 [17.00-35.00] | 0.174 |
| AST, U/L | 26.00 [20.00-35.00] | 31.00 [20.50-44.50] | 31.00 [22.00-40.00] | 26.00 [20.00-35.00] | <0.001 |
| Total bilirubin, mg/dL | 0.70 [0.50-0.80] | 0.50 [0.45-1.65] | 0.70 [0.50-1.10] | 0.70 [0.50-0.80] | 0.009 |
| GNRI | 101.26 [96.79-104.24] | 77.46 [74.32-79.52] | 93.81 [90.02-96.74] | 101.77 [100.50-105.73] | <0.001 |
| PNI | 48.75 ± 5.68 | 34.57 ± 3.76 | 44.12 ± 4.79 | 50.85 ± 4.21 | <0.001 |
| HALP | 48.93 [33.25-67.36] | 11.96 [7.44-17.29] | 36.79 [23.28-55.34] | 54.25 [39.88-71.10] | <0.001 |
| SII | 338.15 [220.00-539.43] | 1319.74 [1065.52-2499.81] | 379.68 [227.16-719.24] | 321.30 [217.38-463.03] | <0.001 |
| ALI | 56.30 ± 28.29 | 12.37 ± 8.75 | 41.80 ± 23.60 | 62.85 ± 27.06 | <0.001 |
| LAR | 1.40 [1.14-1.69] | 2.63 [1.83-3.70] | 1.51 [1.19-2.01] | 1.35 [1.12-1.60] | <0.001 |

Values are presented as mean ± standard deviation or median IQR, as appropriate. GNRI categories were defined as <82, 82 to <98, and ≥98. eGFR, estimated glomerular filtration rate; ALT, alanine aminotransferase; AST, aspartate aminotransferase; GNRI, Geriatric Nutritional Risk Index; PNI, Prognostic Nutritional Index; HALP, hemoglobin, albumin, lymphocyte, and platelet index; SII, systemic immune-inflammation index; ALI, advanced lung cancer inflammation index; LAR, leukocyte-to-albumin ratio; IQR, interquartile range; SD, standard deviation.

**Table S2.** Missingness of variables included in the primary and sensitivity analyses.

| Variable | Missing, n | Missing, % |
| --- | --- | --- |
| **Main-analysis variables** |  |  |
| Postoperative ICU admission | 0 | 0 |
| GNRI | 0 | 0 |
| PNI | 0 | 0 |
| HALP | 0 | 0 |
| SII | 0 | 0 |
| ALI | 0 | 0 |
| LAR | 0 | 0 |
| Age | 0 | 0 |
| BMI | 0 | 0 |
| Creatinine | 24 | 2.01 |
| ASA | 18 | 1.51 |
| **Sensitivity-analysis variables** |  |  |
| ALT | 104 | 8.7 |
| AST | 104 | 8.7 |
| Total bilirubin | 106 | 8.87 |
| eGFR | 24 | 2.01 |
| Operative duration hours | 0 | 0 |
| Preoperative GNRI (within 3 days before surgery) | 104 | 8.7 |
| Preoperative PNI (within 3 days before surgery) | 113 | 9.46 |
| Preoperative HALP (within 3 days before surgery) | 113 | 9.46 |
| Preoperative SII (within 3 days before surgery) | 107 | 8.95 |
| Preoperative ALI (within 3 days before surgery) | 113 | 9.46 |
| Preoperative LAR (within 3 days before surgery) | 109 | 9.12 |

Missingness was assessed in the original cohort before multiple imputation (n = 1,195), and percentages used the total cohort as the denominator. The postoperative ICU admission outcome was not imputed. Preoperative 3-day indices were derived from the first available laboratory measurements obtained within 3 days before surgery.

**Table S3.** Sensitivity analysis of the associations between nutritional and inflammatory indices and postoperative ICU admission.

| **Exposures** | **Non-adjusted** | **Adjusted I** | **Adjusted II** |
| --- | --- | --- | --- |
| **GNRI tertiles** |  |  |  |
| GNRI (per SD change) | 0.564 (0.482-0.660)^***^ | 0.543 (0.450-0.655)^***^ | 0.576 (0.473-0.701)^***^ |
| T1 | 1 (ref) | 1 (ref) | 1 (ref) |
| T2 | 0.364 (0.155-0.852)^*^ | 0.348 (0.141-0.859)^*^ | 0.430 (0.167-1.107) |
| T3 | 0.123 (0.053-0.286)^***^ | 0.117 (0.045-0.301)^***^ | 0.158 (0.059-0.425)^***^ |
| *P* for trend | <0.001 | <0.001 | <0.001 |
| **ALI tertiles** |  |  |  |
| ALI (per SD change) | 0.584 (0.495-0.689)^***^ | 0.614 (0.509-0.740)^***^ | 0.665 (0.547-0.808)^***^ |
| T1 | 1 (ref) | 1 (ref) | 1 (ref) |
| T2 | 0.354 (0.224-0.559)^***^ | 0.387 (0.241-0.621)^***^ | 0.416 (0.253-0.683)^**^ |
| T3 | 0.354 (0.224-0.559)^***^ | 0.429 (0.259-0.712)^**^ | 0.488 (0.287-0.830)^**^ |
| *P* for trend | <0.001 | 0.001 | 0.006 |
| **HALP tertiles** |  |  |  |
| HALP (per SD change) | 0.577 (0.487-0.684)^***^ | 0.603 (0.501-0.726)^***^ | 0.663 (0.546-0.804)^***^ |
| T1 | 1 (ref) | 1 (ref) | 1 (ref) |
| T2 | 0.374 (0.239-0.585)^***^ | 0.410 (0.258-0.652)^***^ | 0.456 (0.280-0.744)^**^ |
| T3 | 0.309 (0.193-0.496)^***^ | 0.341 (0.206-0.564)^***^ | 0.419 (0.247-0.709)^**^ |
| *P* for trend | <0.001 | <0.001 | 0.001 |
| **LAR tertiles** |  |  |  |
| LAR (per SD change) | 1.733 (1.473-2.039)^***^ | 1.600 (1.353-1.893)^***^ | 1.487 (1.246-1.776)^***^ |
| T1 | 1 (ref) | 1 (ref) | 1 (ref) |
| T2 | 1.202 (0.705-2.049) | 1.035 (0.601-1.782) | 0.927 (0.529-1.624) |
| T3 | 3.002 (1.879-4.796)^***^ | 2.398 (1.482-3.880)^***^ | 2.034 (1.232-3.358)^**^ |
| *P* for trend | <0.001 | <0.001 | 0.001 |
| **PNI tertiles** |  |  |  |
| PNI (per SD change) | 0.577 (0.489-0.681)^***^ | 0.616 (0.515-0.736)^***^ | 0.646 (0.535-0.780)^***^ |
| T1 | 1 (ref) | 1 (ref) | 1 (ref) |
| T2 | 0.570 (0.377-0.863)^**^ | 0.626 (0.409-0.959)^*^ | 0.635 (0.406-0.992)^*^ |
| T3 | 0.255 (0.152-0.430)^***^ | 0.314 (0.184-0.538)^***^ | 0.353 (0.202-0.617)^***^ |
| *P* for trend | <0.001 | <0.001 | <0.001 |
| **SII tertiles** |  |  |  |
| SII (per SD change) | 1.733 (1.457-2.062)^***^ | 1.590 (1.325-1.909)^***^ | 1.453 (1.202-1.756)^***^ |
| T1 | 1 (ref) | 1 (ref) | 1 (ref) |
| T2 | 1.209 (0.704-2.079) | 1.151 (0.665-1.990) | 1.028 (0.582-1.815) |
| T3 | 3.235 (2.016-5.190)^***^ | 2.655 (1.630-4.324)^***^ | 2.278 (1.369-3.789)^**^ |
| *P* for trend | <0.001 | <0.001 | <0.001 |

The sensitivity analysis included patients with complete data for the outcome, all six nutritional and inflammatory indices, and the covariates included in the adjusted models. The same complete-case cohort (n = 1,146) was used for the non-adjusted, Adjusted I, and Adjusted II models. GNRI, ALI, HALP, LAR, PNI, and SII were analyzed as continuous variables per standard deviation increase and as tertiles, with T1 as the reference category. The non-adjusted model included no covariates. Adjusted I was adjusted for age, sex, and BMI. Adjusted II was additionally adjusted for ASA group, procedure category, creatinine, chronic liver disease, diabetes, hypertension, and major cardiovascular disease. Results are presented as odds ratios (ORs) with 95% confidence intervals (CIs). *P* for trend was calculated by modeling tertile categories as an ordinal variable. In the complete-case cohort, the ALI T2 and T3 groups each included 382 participants, with 29 postoperative ICU admissions and 353 non-admissions in each group. Their identical unadjusted 2 × 2 outcome distributions therefore resulted in identical unadjusted ORs and 95% CIs. The adjusted estimates differed after covariate adjustment. ^*^*P* < 0.05, ^**^*P* < 0.01, and ^***^*P* < 0.001. BMI, body mass index; ASA, American Society of Anesthesiologists; GNRI, geriatric nutritional risk index; PNI, prognostic nutritional index; HALP, hemoglobin-albumin-lymphocyte-platelet score; SII, systemic immune-inflammation index; ALI, advanced lung cancer inflammation index; LAR, leukocyte-to-albumin ratio.

**Table S4.** Sensitivity analysis of GNRI modeled as a continuous variable and as study-population tertiles.

| **Exposures** | **Non-adjusted** | **Adjusted I** | **Adjusted II** |
| --- | --- | --- | --- |
| **GNRI tertiles** |  |  |  |
| GNRI (per SD change) | 0.581 (0.499-0.676)^***^ | 0.565 (0.471-0.677)^***^ | 0.601 (0.497-0.728)^***^ |
| T1 | 1 (ref) | 1 (ref) | 1 (ref) |
| T2 | 0.439 (0.290-0.662)^***^ | 0.456 (0.293-0.709)^***^ | 0.501 (0.318-0.790)^**^ |
| T3 | 0.247 (0.152-0.402)^***^ | 0.272 (0.162-0.459)^***^ | 0.309 (0.179-0.532)^***^ |
| *P* for trend | <0.001 | <0.001 | <0.001 |

GNRI was analyzed as a continuous variable per standard deviation increase and as study-population tertiles, with T1 as the reference category. The non-adjusted model included no covariates. Adjusted I was adjusted for age, sex, and BMI. Adjusted II was additionally adjusted for ASA group, procedure category, creatinine, chronic liver disease, diabetes, hypertension, and major cardiovascular disease. Results are presented as odds ratios (ORs) with 95% confidence intervals (CIs). *P* for trend was calculated by modeling tertile categories as an ordinal variable. Model estimates were pooled across 20 completed datasets using Rubin's rules. ^*^*P* < 0.05, ^**^*P* < 0.01, and ^***^*P* < 0.001. BMI, body mass index; ASA, American Society of Anesthesiologists; GNRI, Geriatric Nutritional Risk Index.

**Table S5.** Logistic regression sensitivity analyses incorporating liver function, operative duration, and eGFR.

| **Exposures** | **Adjusted III** | **Adjusted IV** | **Adjusted V** |
| --- | --- | --- | --- |
| **GNRI tertiles** |  |  |  |
| GNRI (per SD change) | 0.678 (0.556-0.828)^***^ | 0.667 (0.530-0.839)^**^ | 0.599 (0.495-0.725)^***^ |
| T1 | 1 (ref) | 1 (ref) | 1 (ref) |
| T2 | 0.561 (0.219-1.438) | 0.247 (0.084-0.730)^*^ | 0.538 (0.213-1.357) |
| T3 | 0.269 (0.101-0.719)^**^ | 0.147 (0.049-0.444)^**^ | 0.208 (0.079-0.545)^**^ |
| *P* for trend | 0.001 | <0.001 | <0.001 |
| **ALI tertiles** |  |  |  |
| ALI (per SD change) | 0.737 (0.608-0.893)^**^ | 0.757 (0.606-0.944)^*^ | 0.673 (0.558-0.811)^***^ |
| T1 | 1 (ref) | 1 (ref) | 1 (ref) |
| T2 | 0.579 (0.358-0.935)^*^ | 0.723 (0.427-1.224) | 0.487 (0.306-0.774)^**^ |
| T3 | 0.576 (0.339-0.979)^*^ | 0.545 (0.300-0.991)^*^ | 0.469 (0.279-0.788)^**^ |
| *P* for trend | 0.036 | 0.044 | 0.003 |
| **HALP tertiles** |  |  |  |
| HALP (per SD change) | 0.714 (0.589-0.865)^**^ | 0.796 (0.639-0.990)^*^ | 0.663 (0.549-0.800)^***^ |
| T1 | 1 (ref) | 1 (ref) | 1 (ref) |
| T2 | 0.540 (0.335-0.871)^*^ | 0.604 (0.355-1.028) | 0.469 (0.294-0.747)^**^ |
| T3 | 0.419 (0.246-0.713)^**^ | 0.462 (0.252-0.845)^*^ | 0.377 (0.225-0.633)^***^ |
| *P* for trend | 0.001 | 0.010 | <0.001 |
| **LAR tertiles** |  |  |  |
| LAR (per SD change) | 1.333 (1.119-1.589)^**^ | 1.316 (1.077-1.607)^**^ | 1.433 (1.208-1.700)^***^ |
| T1 | 1 (ref) | 1 (ref) | 1 (ref) |
| T2 | 1.204 (0.698-2.077) | 0.847 (0.462-1.552) | 1.147 (0.673-1.955) |
| T3 | 1.942 (1.175-3.211)^*^ | 1.462 (0.838-2.550) | 2.062 (1.264-3.364)^**^ |
| *P* for trend | 0.005 | 0.076 | 0.001 |
| **PNI tertiles** |  |  |  |
| PNI (per SD change) | 0.728 (0.602-0.881)^**^ | 0.735 (0.590-0.915)^**^ | 0.647 (0.539-0.777)^***^ |
| T1 | 1 (ref) | 1 (ref) | 1 (ref) |
| T2 | 0.741 (0.474-1.158) | 0.875 (0.529-1.447) | 0.624 (0.406-0.959)^*^ |
| T3 | 0.432 (0.247-0.756)^**^ | 0.547 (0.297-1.009) | 0.331 (0.192-0.570)^***^ |
| *P* for trend | 0.003 | 0.066 | <0.001 |
| **SII tertiles** |  |  |  |
| SII (per SD change) | 1.345 (1.118-1.618)^**^ | 1.240 (1.002-1.534)^*^ | 1.432 (1.194-1.718)^***^ |
| T1 | 1 (ref) | 1 (ref) | 1 (ref) |
| T2 | 1.354 (0.778-2.355) | 1.409 (0.761-2.610) | 1.318 (0.765-2.271) |
| T3 | 2.193 (1.313-3.662)^**^ | 2.023 (1.134-3.611)^*^ | 2.401 (1.453-3.967)^**^ |
| *P* for trend | 0.002 | 0.016 | <0.001 |

All six indices were analyzed as continuous variables per 1-SD increase and as tertiles, with T1 as the reference. Adjusted I was adjusted for age, sex, and BMI. Adjusted II was additionally adjusted for ASA group, procedure category, creatinine, chronic liver disease, diabetes, hypertension, and major cardiovascular disease. Adjusted III denotes the liver-function sensitivity model, defined as Adjusted II plus preoperative ALT, AST, and total bilirubin. Adjusted IV denotes the operative-duration sensitivity model, defined as Adjusted II plus operative duration. Adjusted V replaced preoperative creatinine with eGFR in Adjusted II. *P* for trend was obtained by modeling tertile categories as an ordinal variable. Results are presented as ORs with 95% CIs. Models III-V were fitted separately across 20 completed datasets and pooled using Rubin's rules. ^*^*P* < 0.05, ^**^*P* < 0.01, ^***^*P* < 0.001. BMI, body mass index; ASA, American Society of Anesthesiologists; GNRI, geriatric nutritional risk index; PNI, prognostic nutritional index; HALP, hemoglobin-albumin-lymphocyte-platelet score; SII, systemic immune-inflammation index; ALI, advanced lung cancer inflammation index; LAR, leukocyte-to-albumin ratio.

**Table S6.** Sensitivity analysis using the first laboratory measurements obtained within 3 days before surgery.

| **Exposures** | **Non-adjusted** | **Adjusted I** | **Adjusted II** |
| --- | --- | --- | --- |
| **GNRI tertiles** |  |  |  |
| GNRI (per SD change) | 0.574 (0.487-0.676)^***^ | 0.569 (0.467-0.693)^***^ | 0.599 (0.488-0.737)^***^ |
| T1 | 1 (ref) | 1 (ref) | 1 (ref) |
| T2 | 0.326 (0.136-0.779)^*^ | 0.345 (0.135-0.882)^*^ | 0.380 (0.143-1.011) |
| T3 | 0.112 (0.048-0.264)^***^ | 0.114 (0.043-0.306)^***^ | 0.140 (0.050-0.391)^***^ |
| *P* for trend | <0.001 | <0.001 | <0.001 |
| **ALI tertiles** |  |  |  |
| ALI (per SD change) | 0.568 (0.476-0.678)^***^ | 0.600 (0.490-0.735)^***^ | 0.643 (0.520-0.795)^***^ |
| T1 | 1 (ref) | 1 (ref) | 1 (ref) |
| T2 | 0.372 (0.231-0.599)^***^ | 0.422 (0.257-0.691)^**^ | 0.441 (0.262-0.742)^**^ |
| T3 | 0.313 (0.189-0.517)^***^ | 0.400 (0.229-0.696)^**^ | 0.458 (0.256-0.819)^**^ |
| *P* for trend | <0.001 | 0.001 | 0.006 |
| **HALP tertiles** |  |  |  |
| HALP (per SD change) | 0.582 (0.487-0.695)^***^ | 0.621 (0.511-0.755)^***^ | 0.673 (0.548-0.826)^***^ |
| T1 | 1 (ref) | 1 (ref) | 1 (ref) |
| T2 | 0.394 (0.246-0.633)^***^ | 0.441 (0.270-0.719)^**^ | 0.475 (0.284-0.793)^**^ |
| T3 | 0.319 (0.193-0.528)^***^ | 0.362 (0.213-0.616)^***^ | 0.451 (0.259-0.785)^**^ |
| *P* for trend | <0.001 | <0.001 | 0.003 |
| **LAR tertiles** |  |  |  |
| LAR (per SD change) | 1.701 (1.433-2.018)^***^ | 1.583 (1.326-1.888)^***^ | 1.483 (1.231-1.788)^***^ |
| T1 | 1 (ref) | 1 (ref) | 1 (ref) |
| T2 | 1.233 (0.695-2.186) | 1.115 (0.623-1.998) | 0.982 (0.538-1.791) |
| T3 | 3.115 (1.883-5.154)^***^ | 2.563 (1.530-4.293)^***^ | 2.237 (1.309-3.824)^**^ |
| *P* for trend | <0.001 | <0.001 | <0.001 |
| **PNI tertiles** |  |  |  |
| PNI (per SD change) | 0.577 (0.484-0.687)^***^ | 0.623 (0.514-0.755)^***^ | 0.648 (0.530-0.792)^***^ |
| T1 | 1 (ref) | 1 (ref) | 1 (ref) |
| T2 | 0.552 (0.354-0.860)^**^ | 0.610 (0.386-0.962)^*^ | 0.612 (0.380-0.984)^*^ |
| T3 | 0.261 (0.150-0.452)^***^ | 0.321 (0.182-0.567)^***^ | 0.363 (0.202-0.651)^**^ |
| *P* for trend | <0.001 | <0.001 | <0.001 |
| **SII tertiles** |  |  |  |
| SII (per SD change) | 1.742 (1.449-2.093)^***^ | 1.579 (1.304-1.913)^***^ | 1.457 (1.192-1.782)^***^ |
| T1 | 1 (ref) | 1 (ref) | 1 (ref) |
| T2 | 0.915 (0.514-1.627) | 0.882 (0.493-1.578) | 0.779 (0.426-1.424) |
| T3 | 2.783 (1.717-4.512)^***^ | 2.260 (1.373-3.720)^**^ | 1.946 (1.152-3.287)^*^ |
| *P* for trend | <0.001 | <0.001 | 0.001 |

The six nutritional and inflammatory indices were recalculated using the first available laboratory measurements obtained within 3 days before surgery. This complete-case sensitivity analysis included 1,040 participants, including 114 postoperative ICU admissions. The non-adjusted model included no covariates. Adjusted I was adjusted for age, sex, and BMI. Adjusted II was additionally adjusted for ASA group, procedure category, creatinine, chronic liver disease, diabetes, hypertension, and major cardiovascular disease. Continuous indices were analyzed per 1-SD increase, and tertile analyses used T1 as the reference category. *P* for trend was calculated by modeling tertile categories as an ordinal variable. Results are presented as odds ratios (ORs) with 95% confidence intervals (CIs). ^*^*P* < 0.05, ^**^*P* < 0.01, and ^***^*P* < 0.001. BMI, body mass index; ASA, American Society of Anesthesiologists; GNRI, geriatric nutritional risk index; PNI, prognostic nutritional index; HALP, hemoglobin-albumin-lymphocyte-platelet score; SII, systemic immune-inflammation index; ALI, advanced lung cancer inflammation index; LAR, leukocyte-to-albumin ratio.

**Table S7.** Subgroup interaction *P* values before and after false discovery rate correction.

| Subgroup | *P* for interaction | FDR-adjusted *P* for interaction |
| --- | --- | --- |
| **GNRI** |  |  |
| Age | 0.014 | 0.543 |
| Sex | 0.226 | 0.543 |
| BMI | 0.059 | 0.543 |
| ASA | 0.400 | 0.635 |
| Procedure category | 0.241 | 0.543 |
| Chronic liver disease | 0.032 | 0.543 |
| Diabetes | 0.800 | 0.882 |
| Hypertension | 0.924 | 0.942 |
| Major cardiovascular disease | 0.616 | 0.800 |
| **PNI** |  |  |
| Age | 0.065 | 0.543 |
| Sex | 0.234 | 0.543 |
| BMI | 0.733 | 0.877 |
| ASA | 0.709 | 0.877 |
| Procedure category | 0.763 | 0.877 |
| Chronic liver disease | 0.286 | 0.543 |
| Diabetes | 0.266 | 0.543 |
| Hypertension | 0.747 | 0.877 |
| Major cardiovascular disease | 0.888 | 0.922 |
| **HALP** |  |  |
| Age | 0.108 | 0.543 |
| Sex | 0.621 | 0.800 |
| BMI | 0.622 | 0.800 |
| ASA | 0.753 | 0.877 |
| Procedure category | 0.178 | 0.543 |
| Chronic liver disease | 0.606 | 0.800 |
| Diabetes | 0.144 | 0.543 |
| Hypertension | 0.263 | 0.543 |
| Major cardiovascular disease | 0.418 | 0.646 |
| **SII** |  |  |
| Age | 0.302 | 0.543 |
| Sex | 0.400 | 0.635 |
| BMI | 0.588 | 0.800 |
| ASA | 0.175 | 0.543 |
| Procedure category | 0.221 | 0.543 |
| Chronic liver disease | 0.524 | 0.765 |
| Diabetes | 0.122 | 0.543 |
| Hypertension | 0.845 | 0.894 |
| Major cardiovascular disease | 0.170 | 0.543 |
| **ALI** |  |  |
| Age | 0.182 | 0.543 |
| Sex | 0.174 | 0.543 |
| BMI | 0.973 | 0.973 |
| ASA | 0.206 | 0.543 |
| Procedure category | 0.144 | 0.543 |
| Chronic liver disease | 0.163 | 0.543 |
| Diabetes | 0.116 | 0.543 |
| Hypertension | 0.513 | 0.765 |
| Major cardiovascular disease | 0.282 | 0.543 |
| **LAR** |  |  |
| Age | 0.297 | 0.543 |
| Sex | 0.223 | 0.543 |
| BMI | 0.245 | 0.543 |
| ASA | 0.335 | 0.584 |
| Procedure category | 0.362 | 0.611 |
| Chronic liver disease | 0.023 | 0.543 |
| Diabetes | 0.838 | 0.894 |
| Hypertension | 0.783 | 0.880 |
| Major cardiovascular disease | 0.263 | 0.543 |

Subgroup analyses modeled each index as a continuous variable per 1-SD increase and were adjusted for the covariates in Adjusted II, except for the subgroup-defining variable. Interaction P values used Rubin-pooled Wald tests for a single interaction coefficient or MI-pooled D1 joint Wald tests for multiple coefficients. False discovery rate (FDR)-adjusted *P* values were calculated using the Benjamini-Hochberg procedure across all 54 interaction tests. Subgroups were defined as age <65 versus ≥65 years, male versus female sex, BMI <24 versus ≥24 kg/m^2^, ASA 1-2 versus ≥3, procedure category (liver, gallbladder, or pancreas), chronic liver disease, diabetes, hypertension, and major cardiovascular disease.

**Table S8.** Incremental predictive performance of the clinical model after adding individual continuous nutritional and inflammatory indices.

| **Model** | **AUC (95% CI)** | **Delta AUC** | **Brier score (95% CI)** | **Rank** |
| --- | --- | --- | --- | --- |
| Clinical model | 0.766 (0.728-0.805) | Reference | 0.094 (0.082-0.107) | - |
| Clinical model + GNRI | 0.800 (0.764-0.835) | 0.033 | 0.092 (0.081-0.104) | 1 |
| Clinical model + PNI | 0.787 (0.750-0.825) | 0.021 | 0.092 (0.081-0.104) | 2 |
| Clinical model + HALP | 0.786 (0.749-0.822) | 0.020 | 0.093 (0.082-0.105) | 4 |
| Clinical model + SII | 0.783 (0.746-0.821) | 0.017 | 0.093 (0.081-0.105) | 6 |
| Clinical model + ALI | 0.785 (0.748-0.822) | 0.018 | 0.093 (0.081-0.105) | 5 |
| Clinical model + LAR | 0.787 (0.750-0.824) | 0.021 | 0.093 (0.080-0.105) | 3 |

Each nutritional or inflammatory index was added separately to the same clinical model. For each of the 20 completed datasets, AUC and Brier score were calculated separately; reported point estimates are arithmetic means across imputations. The 95% CIs for AUC and Brier score were obtained from 1,000 multiple-imputation-aware bootstrap resamples. In each resample, participants were sampled from the original incomplete cohort, MICE was repeated to generate 20 completed datasets, and the models were refitted. Delta AUC is the extended-model AUC minus the clinical-model AUC. Model contrasts used paired bootstrap resamples. The clinical model included age, sex, BMI, ASA group, procedure category, creatinine, chronic liver disease, diabetes, hypertension, and major cardiovascular disease. BMI, body mass index; ASA, American Society of Anesthesiologists; AUC, area under the receiver operating characteristic curve.

**Table S9.** Incremental reclassification and discrimination improvement after adding individual continuous nutritional and inflammatory indices to the clinical model.

| **Model** | **NRI (95% CI)** | **IDI (95% CI)** |
| --- | --- | --- |
| Clinical model |  |  |
| Clinical model + GNRI | 0.312 (0.139-0.487) | 0.028 (0.013-0.045) |
| Clinical model + PNI | 0.301 (0.128-0.469) | 0.024 (0.010-0.039) |
| Clinical model + HALP | 0.219 (0.047-0.385) | 0.020 (0.009-0.032) |
| Clinical model + SII | 0.225 (0.049-0.386) | 0.015 (0.004-0.025) |
| Clinical model + ALI | 0.355 (0.185-0.520) | 0.018 (0.006-0.030) |
| Clinical model + LAR | 0.184 (0.009-0.360) | 0.017 (0.005-0.029) |

Each nutritional or inflammatory index was added separately to the same clinical model. Continuous NRI and IDI were calculated within each of the 20 completed datasets, and reported point estimates are arithmetic means across imputations. The 95% CIs were obtained from 1,000 multiple-imputation-aware bootstrap resamples: participants were sampled from the original incomplete cohort, MICE was repeated to generate 20 completed datasets, and both models were refitted in each resample. The clinical model included age, sex, BMI, ASA group, procedure category, creatinine, chronic liver disease, diabetes, hypertension, and major cardiovascular disease. BMI, body mass index; ASA, American Society of Anesthesiologists; NRI, net reclassification improvement; IDI, integrated discrimination improvement.

**Table S10.** Sensitivity analysis of the incremental predictive performance of the clinical model after adding individual continuous nutritional and inflammatory indices.

| **Model** | **AUC (95% CI)** | **Delta AUC** | **Brier score (95% CI)** | **Rank** |
| --- | --- | --- | --- | --- |
| Clinical model | 0.769 (0.729-0.810) | Reference | 0.091 (0.079-0.105) | - |
| Clinical model + GNRI | 0.803 (0.765-0.840) | 0.033 | 0.088 (0.076-0.100) | 1 |
| Clinical model + PNI | 0.788 (0.748-0.828) | 0.018 | 0.088 (0.076-0.100) | 3 |
| Clinical model + HALP | 0.788 (0.749-0.826) | 0.018 | 0.089 (0.076-0.101) | 4 |
| Clinical model + SII | 0.786 (0.747-0.825) | 0.017 | 0.090 (0.077-0.103) | 6 |
| Clinical model + ALI | 0.787 (0.747-0.826) | 0.017 | 0.089 (0.078-0.102) | 5 |
| Clinical model + LAR | 0.792 (0.754-0.831) | 0.023 | 0.089 (0.077-0.102) | 2 |

The sensitivity analysis included 1,146 patients, including 130 postoperative ICU admissions. Each nutritional or inflammatory index was added separately as a continuous variable to the same clinical model. The clinical model included age, sex, BMI, ASA group, procedure category, creatinine, chronic liver disease, diabetes, hypertension, and major cardiovascular disease. BMI, body mass index; ASA, American Society of Anesthesiologists; AUC, area under the curve; GNRI, geriatric nutritional risk index; PNI, prognostic nutritional index; HALP, hemoglobin-albumin-lymphocyte-platelet score; SII, systemic immune-inflammation index; ALI, advanced lung cancer inflammation index; LAR, leukocyte-to-albumin ratio.

**Table S11.** Sensitivity analysis of incremental reclassification and discrimination improvement after adding individual continuous nutritional and inflammatory indices to the clinical model.

| **Model** | **NRI (95% CI)** | **IDI (95% CI)** |
| --- | --- | --- |
| Clinical model |  |  |
| Clinical model + GNRI | 0.364 (0.195-0.557) | 0.035 (0.017-0.055) |
| Clinical model + PNI | 0.302 (0.118-0.490) | 0.026 (0.011-0.042) |
| Clinical model + HALP | 0.210 (0.028-0.396) | 0.021 (0.007-0.034) |
| Clinical model + SII | 0.221 (0.034-0.402) | 0.017 (0.006-0.029) |
| Clinical model + ALI | 0.362 (0.171-0.532) | 0.019 (0.006-0.032) |
| Clinical model + LAR | 0.213 (0.036-0.408) | 0.021 (0.008-0.035) |

The sensitivity analysis included 1,146 patients, including 130 postoperative ICU admissions. Each nutritional or inflammatory index was added separately as a continuous variable to the same clinical model. The clinical model included age, sex, BMI, ASA group, procedure category, creatinine, chronic liver disease, diabetes, hypertension, and major cardiovascular disease. BMI, body mass index; ASA, American Society of Anesthesiologists; NRI, net reclassification improvement; IDI, integrated discrimination improvement; GNRI, geriatric nutritional risk index; PNI, prognostic nutritional index; HALP, hemoglobin-albumin-lymphocyte-platelet score; SII, systemic immune-inflammation index; ALI, advanced lung cancer inflammation index; LAR, leukocyte-to-albumin ratio.

**Table S12.** Bootstrap internal validation and optimism-corrected AUC of the prediction models.

| Model | Apparent AUC | Mean optimism | Optimism-corrected AUC (95% CI) | Calibration slope |
| --- | --- | --- | --- | --- |
| Clinical model | 0.766 | 0.022 | 0.744 (0.707-0.783) | 0.878 |
| Clinical model + GNRI | 0.800 | 0.020 | 0.780 (0.744-0.816) | 0.897 |
| Clinical model + PNI | 0.787 | 0.021 | 0.767 (0.731-0.805) | 0.891 |
| Clinical model + HALP | 0.786 | 0.022 | 0.764 (0.724-0.800) | 0.885 |
| Clinical model + SII | 0.783 | 0.022 | 0.761 (0.725-0.799) | 0.886 |
| Clinical model + ALI | 0.785 | 0.022 | 0.763 (0.725-0.801) | 0.887 |
| Clinical model + LAR | 0.787 | 0.021 | 0.766 (0.731-0.802) | 0.889 |

The point estimates of optimism-corrected AUC and calibration slope were obtained by bootstrap internal validation across the 20 imputed datasets, using 1,000 inner bootstrap resamples with the same resampling indices applied across imputations. Mean optimism was the mean difference between bootstrap-sample AUC and test-sample AUC. Optimism-corrected AUC was calculated as the mean apparent AUC minus mean optimism across imputations. The 95% CIs were obtained from 500 outer bootstrap resamples of the original incomplete cohort. MICE was repeated to generate 20 datasets in each outer resample, and the optimism correction was repeated using 100 inner bootstrap resamples. The reported calibration slope is the mean bootstrap validation slope and serves as an estimate of the global shrinkage factor. AUC, area under the receiver operating characteristic curve.

**Table S13.** Incremental predictive performance of GNRI components and GNRI.

| **Model** | **AUC (95% CI)** | **Delta AUC** | **Brier score (95% CI)** |
| --- | --- | --- | --- |
| Clinical model | 0.766 (0.728-0.805) | Reference | 0.094 (0.083-0.106) |
| Clinical model + albumin | 0.799 (0.763-0.834) | 0.033 | 0.092 (0.080-0.103) |
| Clinical model + weight ratio | 0.768 (0.730-0.807) | 0.002 | 0.095 (0.083-0.108) |
| Clinical model + albumin + weight ratio | 0.799 (0.764-0.835) | 0.033 | 0.092 (0.080-0.104) |
| Clinical model + GNRI | 0.800 (0.764-0.835) | 0.033 | 0.092 (0.080-0.104) |

The clinical model included age, sex, BMI, ASA group, procedure category, preoperative creatinine, chronic liver disease, diabetes, hypertension, and major cardiovascular disease. Albumin and the GNRI weight ratio were added separately and jointly where indicated. AUC and Brier score were calculated within each of the 20 completed datasets. Point estimates are arithmetic means across imputations. Their 95% CIs were obtained from 1,000 multiple-imputation-aware bootstrap resamples of the original incomplete cohort, with MICE repeated and models refitted in each resample. Delta AUC is the extended-model AUC minus the clinical-model AUC. AUC, area under the receiver operating characteristic curve.

**Table S14.** Continuous NRI and IDI after adding GNRI components to the clinical model.

| **Model** | **NRI (95% CI)** | **IDI (95% CI)** |
| --- | --- | --- |
| Clinical model |  |  |
| Clinical model + albumin | 0.329 (0.158-0.495) | 0.032 (0.015-0.048) |
| Clinical model + weight ratio | 0.019 (-0.151-0.198) | 0.000 (-0.002-0.002) |
| Clinical model + albumin + weight ratio | 0.362 (0.187-0.532) | 0.031 (0.016-0.048) |
| Clinical model + GNRI | 0.312 (0.144-0.481) | 0.028 (0.012-0.044) |

Comparisons were made against the clinical model. NRI was calculated as continuous NRI without prespecified risk categories, and IDI represents the change in discrimination slope. Point estimates are arithmetic means across the 20 completed datasets. The 95% CIs were obtained from 1,000 multiple-imputation-aware bootstrap resamples of the original incomplete cohort. MICE was repeated to generate 20 datasets and both models were refitted in each resample. The GNRI composite was evaluated separately from its components. NRI, net reclassification improvement; IDI, integrated discrimination improvement.

**Table S15.** Incremental predictive performance of individual nutritional and inflammatory indices beyond GNRI.

| **Model** | **AUC (95% CI)** | **Delta AUC** | **Brier score (95% CI)** |
| --- | --- | --- | --- |
| Clinical model + GNRI | 0.800 (0.764-0.835) | Reference | 0.092 (0.080-0.105) |
| Clinical model + GNRI + PNI | 0.798 (0.763-0.834) | -0.001 | 0.092 (0.079-0.104) |
| Clinical model + GNRI + HALP | 0.801 (0.766-0.837) | 0.002 | 0.092 (0.080-0.104) |
| Clinical model + GNRI + SII | 0.806 (0.771-0.841) | 0.006 | 0.092 (0.079-0.103) |
| Clinical model + GNRI + ALI | 0.802 (0.767-0.837) | 0.002 | 0.092 (0.080-0.105) |
| Clinical model + GNRI + LAR | 0.806 (0.771-0.840) | 0.006 | 0.092 (0.080-0.104) |

The clinical model plus GNRI served as the reference model. PNI, HALP, SII, ALI, and LAR were added separately in distinct extended models. AUC and Brier score were calculated within each of the 20 completed datasets. Point estimates are arithmetic means across imputations. Their 95% CIs were obtained from 1,000 multiple-imputation-aware bootstrap resamples of the original incomplete cohort, with MICE repeated and models refitted in each resample. Delta AUC is the extended-model AUC minus the clinical-model-plus-GNRI AUC. Each additional index was evaluated in a separate extended model. AUC, area under the receiver operating characteristic curve.

**Table S16.** Continuous NRI and IDI for individual nutritional and inflammatory indices beyond GNRI.

| **Model** | **NRI (95% CI)** | **IDI (95% CI)** |
| --- | --- | --- |
| Clinical model + GNRI |  |  |
| Clinical model + GNRI + PNI | 0.030 (-0.138-0.215) | 0.001 (-0.001-0.002) |
| Clinical model + GNRI + HALP | 0.048 (-0.128-0.226) | 0.005 (-0.000-0.010) |
| Clinical model + GNRI + SII | 0.038 (-0.139-0.217) | 0.006 (0.000-0.013) |
| Clinical model + GNRI + ALI | 0.110 (-0.068-0.299) | 0.004 (-0.001-0.009) |
| Clinical model + GNRI + LAR | 0.050 (-0.114-0.225) | 0.003 (-0.002-0.008) |

The clinical model plus GNRI served as the reference model. PNI, HALP, SII, ALI, and LAR were added separately in distinct extended models. NRI was calculated as continuous NRI without prespecified risk categories, and IDI represents the change in discrimination slope. Point estimates are arithmetic means across the 20 completed datasets. The 95% CIs were obtained from 1,000 multiple-imputation-aware bootstrap resamples of the original incomplete cohort. MICE was repeated to generate 20 datasets and both models were refitted in each resample. NRI, net reclassification improvement; IDI, integrated discrimination improvement.
